# Unexpected degradation of the bisphosphonate P-C-P bridge under mild conditions

**DOI:** 10.1186/1860-5397-4-7

**Published:** 2008-01-21

**Authors:** Petri A Turhanen, Jouko J Vepsäläinen

**Affiliations:** 1 University of Kuopio, Department of Biosciences, Laboratory of Chemistry, PO Box 1627, FIN-70211, Kuopio, Finland

## Abstract

Unexpected degradation of the P-C-P bridge from novel bisphosphonate derivative **1a** and known etidronate trimethyl ester (**1b**) has been observed under mild reaction conditions. A proposed reaction mechanism for the unexpected degradation of **1a** and **1b** is also reported.

## Background

**Figure 1:**
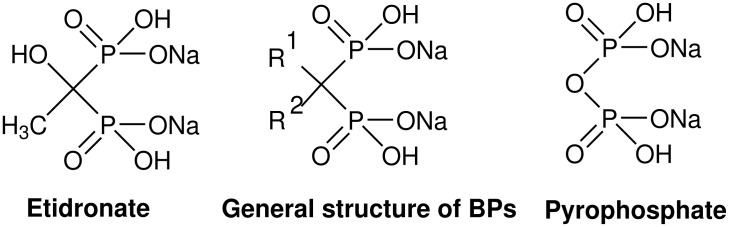
Structures of etidronate, pyrophosphate and general structure of bisphosphonates.

Bisphosphonates (BPs) are analogs of naturally occurring pyrophosphate, where the chemically and enzymatically labile P-O-P bridge has been replaced with a P-C-P bridge, making these compounds relatively resistant to chemical hydrolysis and completely resistant to enzymatic hydrolysis (Figure 1) [1–5]. These BP compounds bind strongly to calcium phosphate and inhibit its formation, aggregation and dissolution [6]. The affinity for the bone mineral represents the basis for their use in the treatment of many diseases associated with increased bone resorption, such as metastatic bone disease, Paget's disease and osteoporosis [1–6]. As described above, the BPs have been used for decades in the therapy of bone diseases but recently these compounds have been found to be active in many other fields, such as in the treatment of parasitic diseases [7–11] and atherosclerosis [12]. Furthermore, the BPs have been shown to be effective against calcifying nanoparticles (CNPs, known also as nanobacteria) which may be responsible for several human diseases where calcium phosphate deposition is a hallmark, e.g. cardiovascular diseases, kidney stones, urological diseases, e.g. prostatitis, many cancers and various forms of autoimmune diseases [13–14]. Therefore it is very important to understand the chemistry of BPs in detail.

**Scheme 1:**
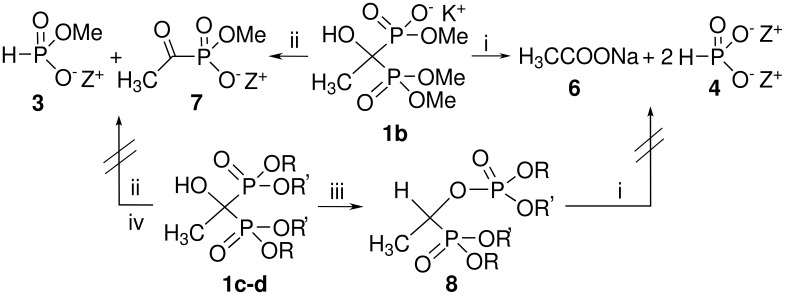
Degradation of trimethyl ester of etidronate (1b) and stability of the tetramethyl ester (**1c**, R=R`=Me) and P,P`-dimethyl ester (**1d**, R=Me, R`=Na^+^) of etidronate. *Reagents and conditions:* i) 1 drop of 6 M NaOH, H_2_O, 1 h, rt, (measured pH was ≥ 11); ii) 5 equiv triethylamine, H_2_O, 1 h, 60 °C; iii) when R=R`=Me (**1c**), 1 eq, triethylamine, H_2_O, 10 min. ca. 98% conversion. iv) when R=Me, R`=Na^+^ (**1d**), 5 equiv NaOH, H_2_O, overnight, reflux.

Etidronate, (1-hydroxyethylidene)-1,1-bisphosphonic acid (HEBPA) disodium salt, is one of the earliest synthesized and is the most extensively investigated BP compound, still being in clinical use today (Figure 1) [1–6,15]. Our group has designed, synthesized and studied *in vitro* several different etidronate and alendronate derivatives to act as biodegradable prodrugs of these drugs [16–23]. During our ongoing study to prepare new, possibly bioreversible BP derivatives, we observed unexpected degradation of the P-C-P bridge under mild reaction conditions in two of the prepared etidronate derivatives. Earlier, Szymczak et. al. [24] have described the formation of H-phosphonate (also known as phosphite) and phosphate components from a phosphonate-phosphate compound (same kind of structure as **8** in Scheme 1) either in CH_3_CN/Et_3_N/H_2_O (v/v) or phosphate buffer, pH 7.4 at 37 °C. Szajnman et. al. [25] has reported loss of two molecules of phosphite in tetraethyl oxirane-2,2-diylbis(phosphonate); however the kind of degradation which we will discuss in this paper has not been previously reported.

## Results and Discussion

**Scheme 2:**
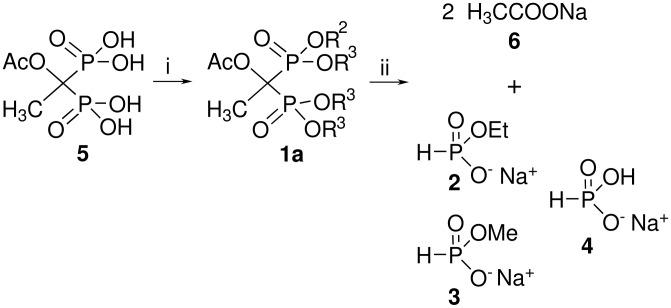
Preparation of BP derivative **1a** (R^2^ = Et, R^3^ = C(O)OEt) and its degradation to acetate **6** and phosphites **2**–**4**. *Reagents and conditions:* i) excess ClC(O)OEt, 6 equiv Na_2_CO_3_, reflux, overnight, 55%; ii) 4 equiv NaOH (40% NaOH in H_2_O), MeOH, 30 min, rt.

As mentioned in the introduction, the P-C-P bridge of BPs has been reported to be relatively stable against chemical hydrolysis, however here we report the unexpectedly easy degradation of two etidronate derivatives into acetate and phosphite moieties. In our ongoing study to prepare novel biodegradable BP derivatives, a new carbonate derivative of etidronate was synthesised. The synthesis was started from the known acetylated etidronic acid [21] (**5**, see Scheme 2) by treating it with ethyl chloroformate and sodium carbonate. The NMR spectroscopy results were surprising since they pointed to the formation of a novel etidronate derivative **1a** (see Scheme 2). In the ^31^P NMR spectrum, there were four doublets (1:1:1:1) due to the presence of two diastereomers. The ^1^H NMR spectrum contained two complicated splitting patterns at approx. 4.46 and 4.28 ppm, their integral ratio was 1 to 3, respectively, indicating two different kinds of -OCH_2_ groups in ratio 1:3. After inspection of ^13^C NMR spectra and the ESI-MS results, we concluded that the prepared molecule had the unanticipated structure of **1a** and not the expected structure where R^2^=R^3^=C(O)OEt (see Scheme 2). To confirm the selective formation of **1a**, the synthesis was repeated several times, but the result was always the same (formation of **1a** was observed in all experiments), though in some experiments a transesterification of the acetyl group to C(O)OEt group was observed in yields of 0–13% as confirmed by the ^1^H and ^31^P NMR spectra. We were unable to provide any direct explanation for the variation in the transesterification proportion. Etidronic acid was also tested as a starting material to prepare a derivative such as **1a** [C(O)OEt group instead of Ac group], but the reaction did not occur under the same conditions as those used in the preparation of **1a**. Our subsequent studies with derivative **1a** led us to another very surprising result, which occurred when 4 equiv of NaOH (40% NaOH in H_2_O) were added to the solution of **1a** in MeOH and stirred for 30 minutes at room temperature. After evaporation of the reaction mixture to dryness, the residue contained almost exclusively (>95% degradation was observed) sodium acetate **6** and phosphites **2**–**4** (compound **4** can be also called phosphorous acid monosodium salt) as can be seen in Scheme 2. Compounds **2**–**4** were readily characterized by their P-H chemical shifts and characteristic ^1^J_HP_ coupling constants (ca. 600 Hz). In the ^31^P NMR spectrum, there were three different monophosphorus components confirmed to be compounds **2**–**4**. Two moles of acetate 6 were detected compared to one mole of the total amount of phosphites **2**–**4** which was the expected result. Interestingly, the decomposition mixture of **1a** contained not only monoethyl phosphite **2** and phosphite **4** but also monomethyl derivative **3** (according the ^31^P NMR spectrum, the ratio was approx.: 1:0.86:1, respectively, see Supporting Information File 1, S13). The formation of this monomethyl phosphite **3** under the conditions used (see Scheme 2, procedure ii) can be explained based on: 1) partial transesterification of bisphosphonate **1a** before degradation of P-C-P bridge, 2) partial esterification of phosphonate group after the carbonate groups (R^3^) decomposition from compound **1a** (this is proposed to occur rapidly after the addition of 40% NaOH) and before the degradation of P-C-P bridge, 3) partial transesterification of **2**, and 4) esterification of **4** (see Scheme 2).

These unexpected degradation results we observed for the HEBPA derivative **1a** led us to examine what would happen to more simple derivatives of HEBPA, such as trimethyl (**1b**), tetramethyl (**1c**) and P,P`-dimethyl (**1d**) esters of etidronate under the same kinds of conditions (see Scheme 1). Compounds **1b**–**d** were prepared as reported elsewhere [20,26–28]. Again very surprising results were obtained. Trimethyl ester of etidronate (**1b**) was degraded to the acetate **6** and phosphorous acid salt **4**, under even milder conditions than the degradation of **1a** (50 mg of **1b** in 1 ml H_2_O and 1 drop of 6 M NaOH was stirred for 1 hour at rt; measured pH was ≥ 11; see Scheme 1). Tetramethyl (**1c**) or P,P`-dimethyl ester were not degraded under the same conditions, only the formation of phosphonate-phosphate derivative **8** from **1c** was observed as expected in the light of the earlier results concern the rearrangement process [17,20–21,29–32]. This rearrangement of **1c** to **8** was observed to happen rapidly and almost completely (98% conversion) when 1 equiv of triethylamine was present in water (see Scheme 1).

Compound **1b** was selectively degraded to the phosphite **3** and acetyl phosphonate **7** when **5** equiv of triethylamine was used in H_2_O (see Scheme 1). Dialkyl acetylphosphonates and dialkyl phosphites are common starting materials for the synthesis of tetraalkyl esters of HEBPA [26–27], but this is the first time when the “reverse” synthesis has been reported. P,P`-dimethyl ester **1d** did not degrade to compounds **3** and **7** or **6** and **4** even when refluxed overnight with 5 equiv NaOH in H_2_O.

**Scheme 3:**
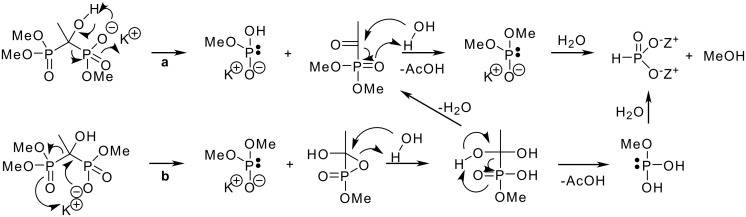
Proposed reaction mechanism for **1b** decomposition.

The decomposition mechanism for **1b** can be explained in two ways; either via a decomposition mechanism resembling the reversible route of the formation of tetraesters (see Scheme 3 route **a**), since e.g., **1c**, are prepared from phosphites, H-P(O)(OMe)_2_, and phosphonates, MeCOP(O)(OMe)_2_, or route **b** resembling the rearrangement process [19]. The driving force in both reactions is the formation of three charged molecules from one P-C-P compound since this is a highly entropically favoured process. Decomposition of the first P-C bond starts with deprotonation of the hydroxyl group followed by elimination of the methyl phosphite and the formation of ketone (route **a**) or by nucleophilic attack of the oxygen of the ionized phosphate on the bridging carbon to release dimethyl phosphite and the oxirane ring containing derivative (route **b**). In route **a**, water or hydroxide ion attacks the carbonyl carbon and P-C bond cleavage occurs giving rise to acetic acid and dimethyl phosphite which can undergo a further reaction with water or hydroxide to give methyl phosphite. In route **b**, the attack of water on the carbon of oxirane ring yields hydrate followed by elimination of methyl phosphite and acetic acid. We believe that route **a** is more probable, since during the reaction with a weaker base, such as triethylamine, only the first P-C bond is cleaved and products **3** and **7** are observed. On the other hand, decomposition of **1a** is more likely to follow route **b**.

**Scheme 4:**
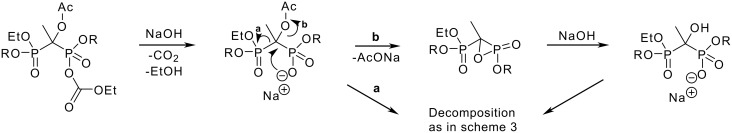
Proposed reaction mechanism for **1a** decomposition.

The proposed decomposition mechanism for **1a** (see Scheme 4) is more complicated. The reaction starts with the hydrolysis of one carbonate ester leading to a monoanion comparable to **1b**. After this step, the decomposition can continue following routes that are similar to either route **a** or route **b** in Scheme 3. The other possibility, route **b** (in Scheme 4), is a nucleophilic attack of oxygen to the bridging carbon and the formation of an oxirane ring containing derivative, since the adjacent acetate group is a rather good leaving group. Subsequently, P-C-bond decomposition will follow the same mechanism as reported in Scheme 3.

The initial reaction in Scheme 3 also explains the formation of rearranged product **8** from tetraester **1c** (this rearrangement is proposed to happen via oxirane ring) [19], since the charged oxygen is a good nucleophile compared to OH-group and far better than oxygen bound to phosphorus with a double bond (P=O).

All of the compounds were easily identified by their ^1^H, ^13^C and ^31^P NMR spectra. In ^31^P NMR signals for the phosphites **2**, **3** and **4** were 6.26 ppm, 8.47 ppm and 5.81 ppm, respectively and 0.10 ppm for acetylphosphonate **7**. These values were comparable to those reported earlier [27].

## Conclusion

In conclusion, a novel carbonate derivative of etidronate (**1a**) was prepared by the reaction of acetylated etidronic acid with ethyl chloroformate and sodium carbonate. Compound **1a** was found to undergo remarkably facile cleavage of the P-C bond under mild basic conditions. The trimethyl ester of etidronate (**1b**) was also found to undergo readily P-C bond cleavage under similar conditions. The trimethyl ester of etidronate (**1b**) was also observed to be degraded to phosphite **3** and acetylphosphonate **7** when mixed in H_2_O containing 5 equiv of triethylamine. Some mechanisms to explain these behaviors have been proposed though further investigations will be necessary to confirm the proposed degradation pathways.

## Supporting Information

File 1 Unexpected degradation of bisphosphonate P-C-P bridge under mild conditions. Experimental procedures, full spectroscopic data and NMR spectra for the novel compound **1a** and NMR spectra for the degradation studies of **1a** and **1b**.
