# Supplementary material for: Unexpected degradation of the bisphosphonate P-C-P bridge under mild conditions
Source: Beilstein J Org Chem. 2008 Jan 21;4:7. doi: 10.1186/1860-5397-4-7 (PMC2238746; doi:10.1186/1860-5397-4-7)
Supplement: File 1 — Unexpected degradation of bisphosphonate P-C-P bridge under mild conditions. Experimental procedures, full spectroscopic data and NMR spectra for the novel compound 1a and NMR spectra for the degradation studies of 1a and 1b. [file Beilstein_J_Org_Chem-04-07-s001.pdf]

Unexpected degradation of bisphosphonate P-C-P bridge under mild conditions

Petri A. Turhanen* and Jouko J. Vepsäläinen

University of Kuopio, Department of Biosciences, Laboratory of Chemistry, P.O. Box 1627, FIN-70211, Kuopio, Finland.

Table of Contents

General experimental details **S2**

Procedure for the preparation of etidronate derivative 1a **S2**

^{1}H, ^{13}C and ^{31}P NMR identification data for compound 1a **S2**

Typical example of 1a degradation experiment **S2**

Typical example of 1b degradation experiment **S2**

Typical example of 1b degradation experiment in triethyl amine **S3**

^{1}H NMR spectra of compound 1a **S4**

^{31}P NMR spectra of compound 1a **S5**

^{13}C NMR spectra with broadens of compound 1a **S6**

Typical examples of ^{1}H and ^{31}P NMR spectra of 1a decomposition experiment **S12**

Typical examples of ^{1}H and ^{31}P NMR spectra of 1b decomposition experiment **S14**

Typical examples of ^{1}H and ^{31}P NMR spectra of 1b decomposition experiment in triethyl amine **S16**

General experimental details: ^{1}H, ^{31}P and ^{13}C NMR spectra were recorded on a Bruker Avance 500 spectrometer operating at 500.1, 202.5 and 125.8 MHz, respectively. TMS or TSP (for D_2O solutions) was used as an internal standard for ^{1}H and ^{13}C measurements, and 85% H_3PO_4 was used as an external standard for ^{31}P measurements. The $^{3}J_{HH}$ couplings are indicated by the letter "*J*". The $^{n}J_{HP}$ couplings were calculated from proton spectra and all *J* values are given in Hz. The $^{n}J_{CP}$ couplings were calculated from proton decoupled carbon spectra with the coupling constants given in parenthesis as hertz. The purity of the compound **1a** was determined from ^{1}H and ^{31}P NMR spectra and was ca. 96 %. Electronspray ionization mass spectra were acquired by an LCQ quadrupole ion trap mass spectrometer with an electronspray ionization source.

Procedure for the preparation of etidronate derivative 1a: Acetylated etidronic acid (**5**) (150 mg, 0.60 mmol), Na_2CO_3 (388 mg, 3.66 mmol) and ethyl chloroformate (3 ml) was refluxed for overnight under the nitrogen atmosphere. Reaction mixture was evaporated to dryness, diethyl ether (8 ml) was added and reaction mixture was stirred for 15 minutes before the precipitate was removed by centrifugation. The remaining solution was evaporated to dryness and compound **1a** was obtained as colorless oil in 55% yield.

[1-(diethoxycarbonyloxy)phosphoryl-1-acetyloxyethyl]-1-(ethoxycarbonyloxy)phosphonic acid ethyl ester (1a): Pair of diastereomers (ratio 50:50). ^{1}H NMR ($CDCl_3$): δ 4.49-4.41 (m, 2H), 4.36-4.18 (m, 6H), 2.14 (s, 3H), 1.973 (dd, $^{3}J_{HP}$ = 15.4, $^{3}J_{HP'}$ = 17.0) and 1.965 (dd, $^{3}J_{HP}$ = 15.5, $^{3}J_{HP'}$ = 17.0, 3H), 1.41-1.34 (m, 12H). ^{13}C NMR ($CDCl_3$) δ 168.91 (dd, $^{3}J_{CP}$ = 7.8, $^{3}J_{CP'}$ = 8.9), 168.76 (dd, $^{3}J_{CP}$ = 7.0, $^{3}J_{CP'}$ = 8.8), 147.67 (d, $^{3}J_{CP}$ = 7.0), 147.66 (d, $^{3}J_{CP}$ = 7.3), 147.59 (d, $^{3}J_{CP}$ = 7.3), 78.79 (dd, $^{1}J_{CP}$ = 156.1, $^{1}J_{CP'}$ = 157.4), 78.71 (dd, $^{1}J_{CP}$ = 155.2, $^{1}J_{CP'}$ = 159.5), 66.27 (d, $^{2}J_{CP}$ = 7.6), 66.14 (d, $^{3}J_{CP}$ = 7.6), 65.82, 65.81, 64.33 (d, $^{4}J_{CP}$ = 6.9), 64.29 (d, $^{4}J_{CP}$ = 7.0), 64.23 (d, $^{4}J_{CP}$ = 7.2), 63.91 (d, $^{4}J_{CP}$ = 7.5), 21.26, 21.24, 18.61 (t, $^{2}J_{CP}$ = 2.8), 18.18 (t, $^{2}J_{CP}$ = 2.0), 16.44 (d, $^{5}J_{CP}$ = 1.8), 16.39 (d, $^{5}J_{CP}$ = 2.6), 16.39 (d, $^{5}J_{CP}$ = 2.6), 16.34 (d, $^{5}J_{CP}$ = 2.6), 15.99 (d, $^{4}J_{CP}$ = 6.7), 13.98, 13.97. ^{31}P NMR ($CDCl_3$) δ 13.19 d ($^{2}J_{PP}$ = 22.5) 10.28 and 13.12 d ($^{2}J_{PP}$ = 21.0) 10.23 d. ESI-MS: 515.3 (M + Na; 100%).

Typical example of 1a degradation experiment: Compound **1a** (70.5 mg, 0.143 mmol) was dissolved in MeOH (2 ml), 40% NaOH (58 µl, 4 eq) was added and the reaction mixture was stirred for 0.5 h at room temperature. Reaction mixture was evaporated to dryness and dried in vacuo. Product (ca. 63 mg) was obtained as white powder and contained mixture of acetate and phosphites **2-4** as identified from ^{1}H and ^{31}P NMR spectra (see pages S12-13).

Typical example of 1b degradation experiment: Compound **1b** (50 mg) was dissolved in H_2O (1 ml), pH was adjusted to ≥ 11 with 6 M NaOH (this needed only 1 pasteur pipette drop) and the reaction mixture was stirred for 1 h at room temperature before evaporating it to dryness in vacuo. Product was obtained as white powder and contained mixture of acetate and phosphite **4** as

identified from ^{1}H and ^{31}P NMR spectra (see pages S14-15; in ^{31}P NMR spectrum the remaining *H*-P coupling can be seen because of insufficient decoupling power).

Typical example of 1b degradation experiment in triethyl amine: Compound **1b** (50 mg) was dissolved in H_2O (1 ml), triethyl amine (122 µl, 5 eq) was added and the reaction mixture was stirred for 1 h at 60 ºC before evaporating it to dryness in vacuo. Product was obtained as slightly yellow solid and contained mixture of acetylphosphonate **7** and phosphite **3** as identified from ^{1}H and ^{31}P NMR spectra (see pages S16-17; in ^{31}P NMR spectrum the remaining *H*-P coupling can be seen for the compound **3** because of insufficient decoupling power).

Compound **1a**

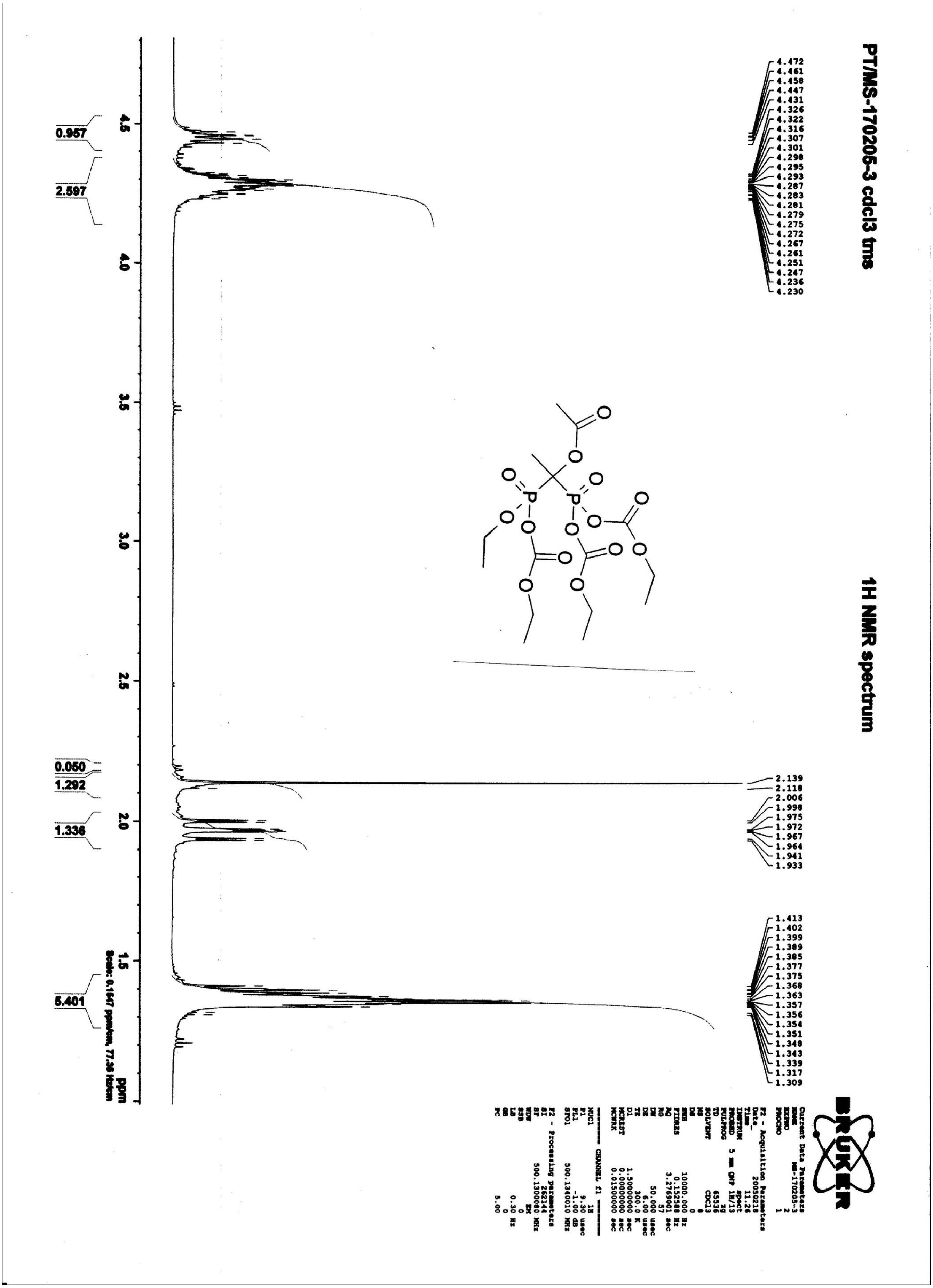

Compound **1a**

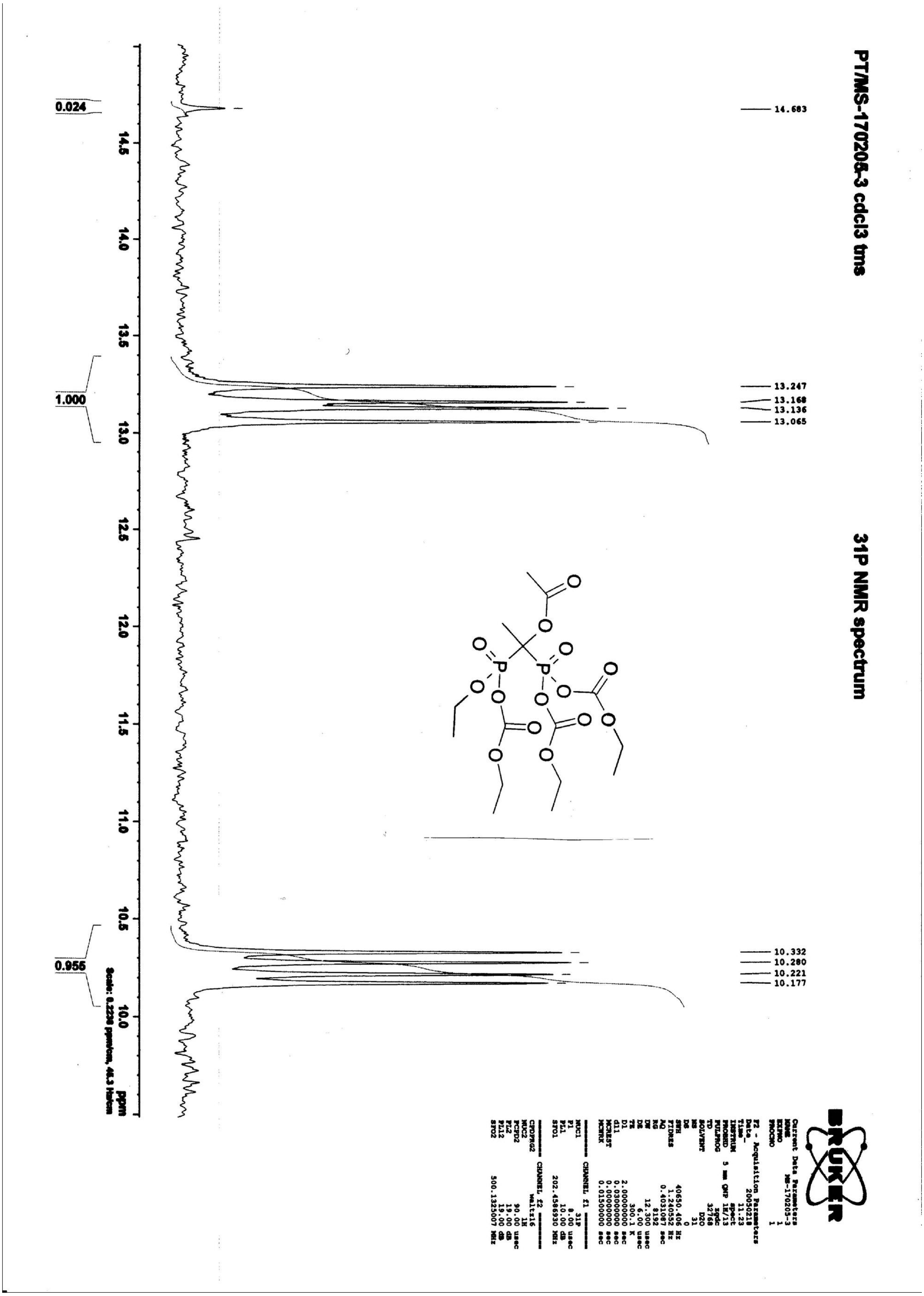

PT/MS-170205-3 cdcl3 tms
31P NMR spectrum
14.683
13.247
13.168
13.136
13.065
10.332
10.280
10.221
10.177
0.024
1.000
0.955
ppm

Compound **1a**

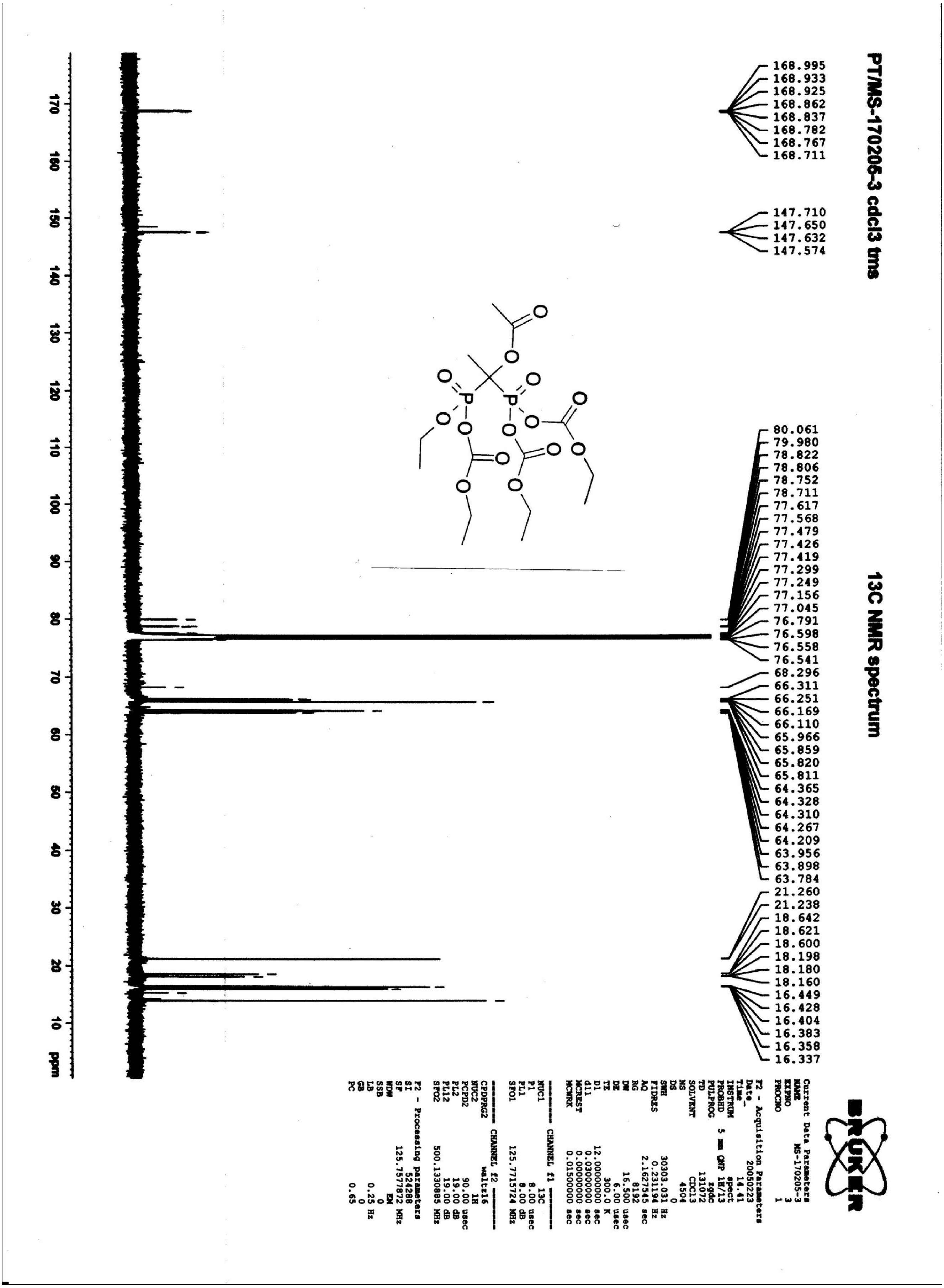
PT/MS-170205-3 cdcl3 tms
13C NMR spectrum
168.995
168.933
168.925
168.862
168.837
168.782
168.767
168.711
147.710
147.650
147.632
147.574
80.061
79.980
78.822
78.806
78.752
78.711
77.617
77.568
77.479
77.426
77.419
77.299
77.249
77.156
77.045
76.791
76.598
76.558
76.541
68.296
66.311
66.251
66.169
66.110
65.966
65.859
65.820
65.811
64.365
64.328
64.310
64.267
64.209
63.956
63.898
63.784
21.260
21.238
18.642
18.621
18.600
18.198
18.180
18.160
16.449
16.428
16.404
16.383
16.358
16.337
Current Data Parameters
NAME MS-170205-3
EXPNO 3
PROCNO 1
F2 - Acquisition Parameters
Date_ 20050223
Time 14.41
INSTRUM spect
PROBHD 5 mm QNP 1H/13
PULPROG zgdc
TD 131072
SOLVENT CDCl3
NS 4504
DS 0
SWH 30303.031 Hz
FIDRES 0.231194 Hz
AQ 2.1627545 sec
RG 8192
DW 16.500 usec
DE 6.00 usec
TE 300.0 K
D1 12.00000000 sec
d11 0.03000000 sec
MCREST 0.00000000 sec
MCWRK 0.01500000 sec
======== CHANNEL f1 ========
NUC1 13C
P1 8.00 usec
PL1 8.00 dB
SFO1 125.7715724 MHz
======== CHANNEL f2 ========
CPDPRG2 waltz16
NUC2 1H
PCPD2 90.00 usec
PL2 19.00 dB
PL12 19.00 dB
SFO2 500.1330885 MHz
F2 - Processing parameters
SI 524288
SF 125.7577872 MHz
WDW EM
SSB 0
LB 0.25 Hz
GB 0
PC 0.65
170
160
150
140
130
120
110
100
90
80
70
60
50
40
30
20
10
ppm

Compound **1a**

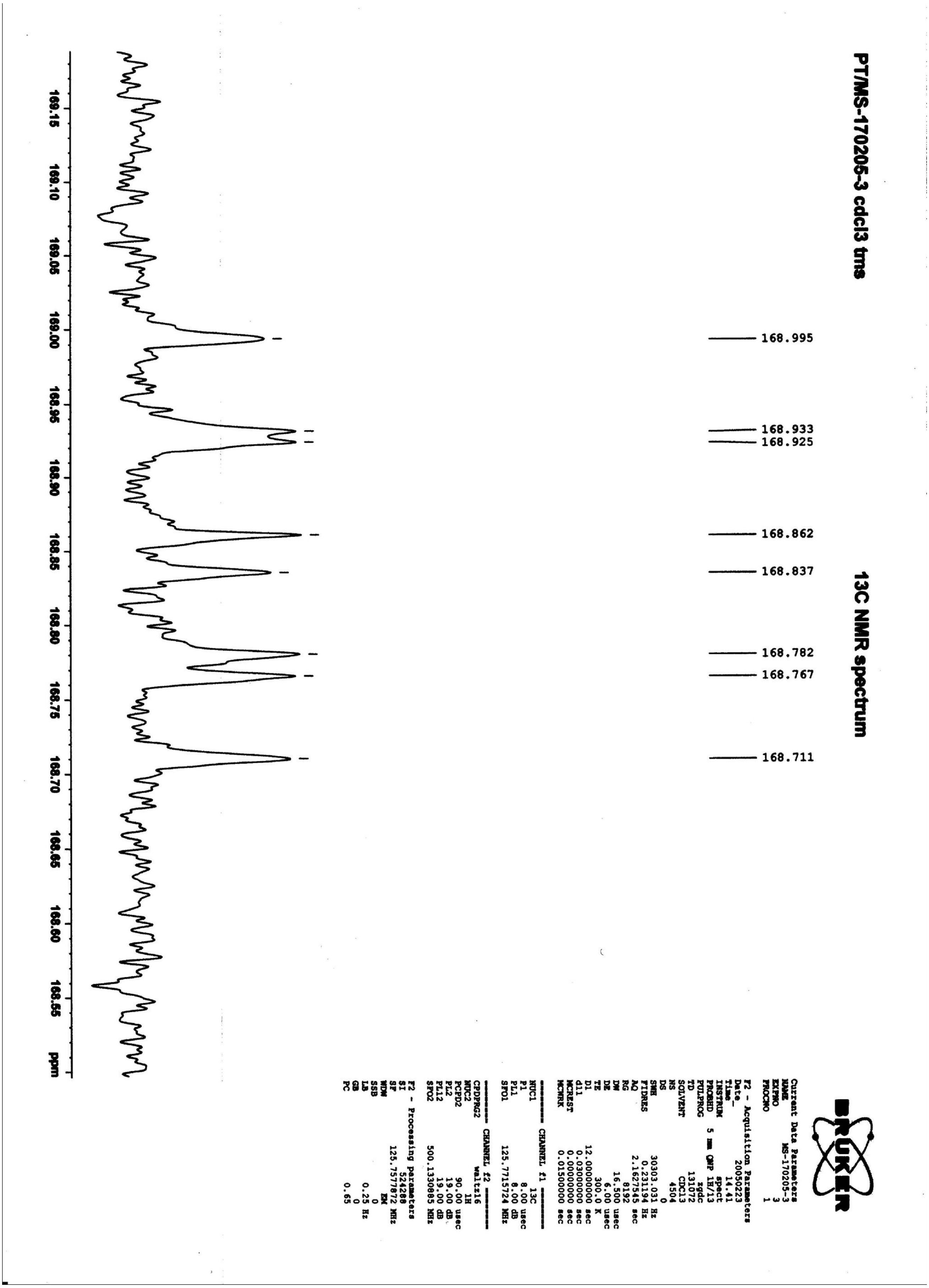

PT/MS-170205-3 cdcl3 tms
13C NMR spectrum
168.995
168.933
168.925
168.862
168.837
168.782
168.767
168.711
ppm

Compound **1a**

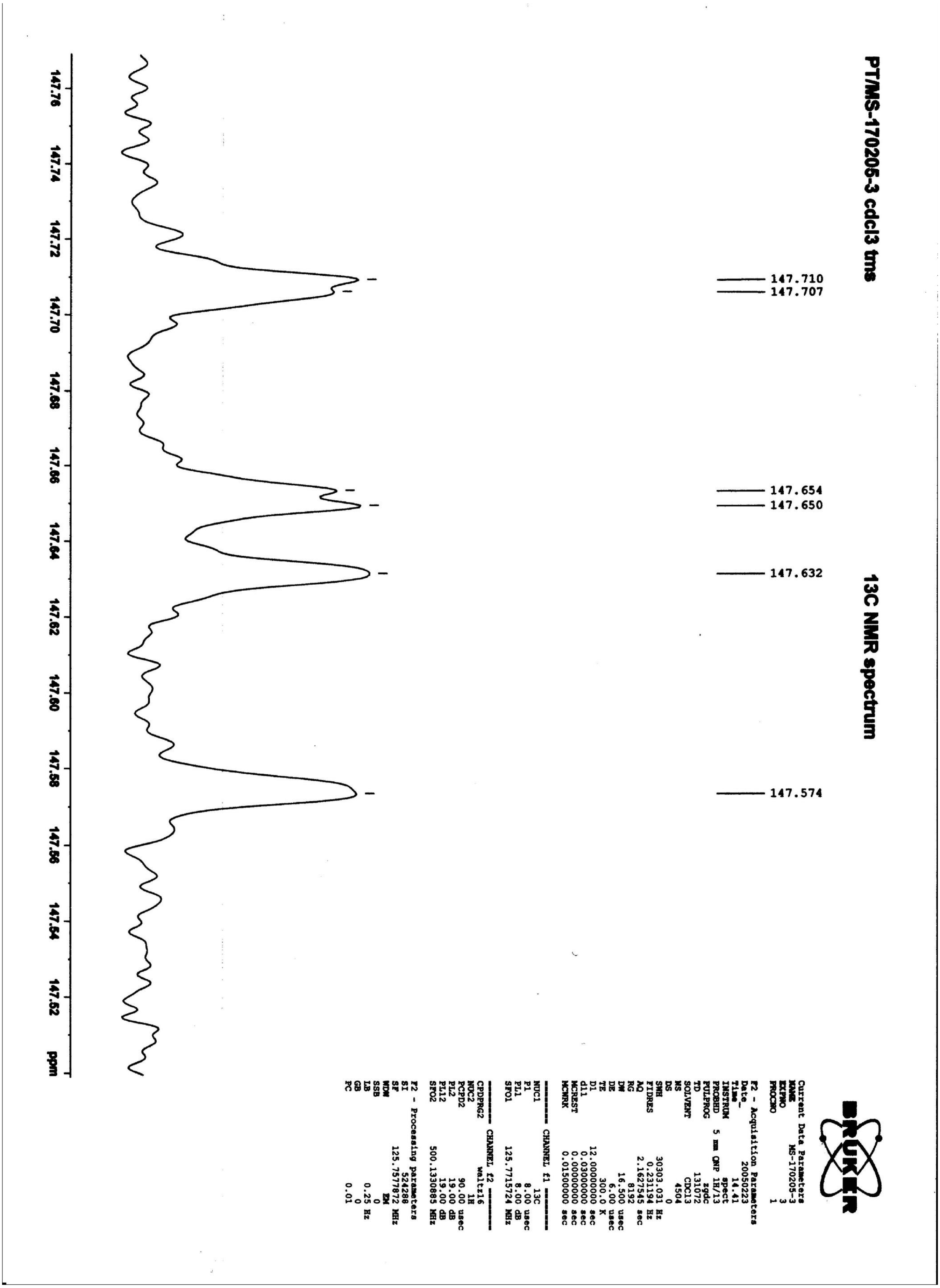

PT/MS-170205-3 cdcl3 tms
13C NMR spectrum
147.710
147.707
147.654
147.650
147.632
147.574
147.76
147.74
147.72
147.70
147.68
147.66
147.64
147.62
147.60
147.58
147.56
147.54
147.52
ppm
BRUKER

Compound **1a**

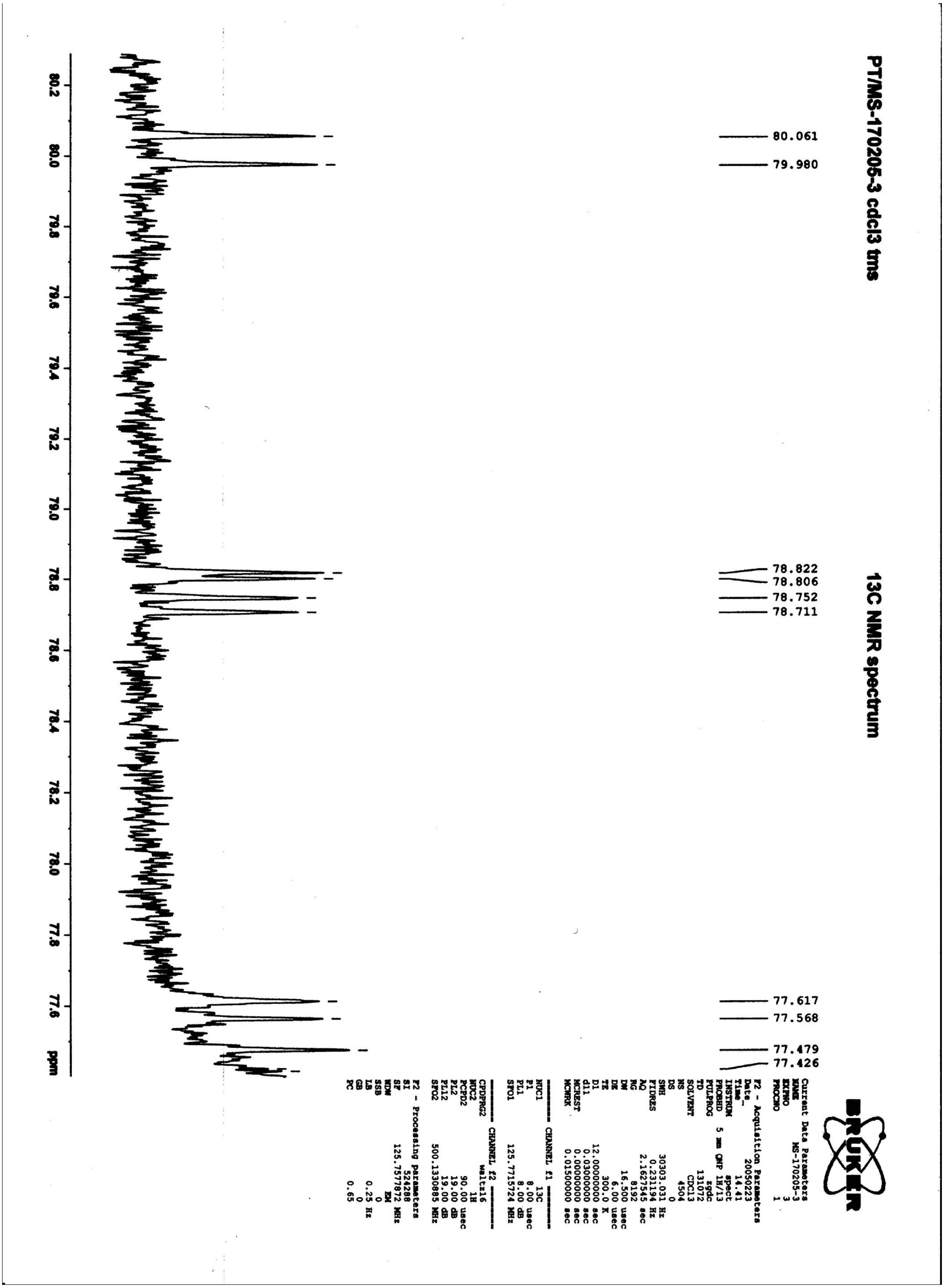
PT/MS-170205-3 cdcl3 tms
13C NMR spectrum
80.061
79.980
78.822
78.806
78.752
78.711
77.617
77.568
77.479
77.426
ppm

Compound **1a**

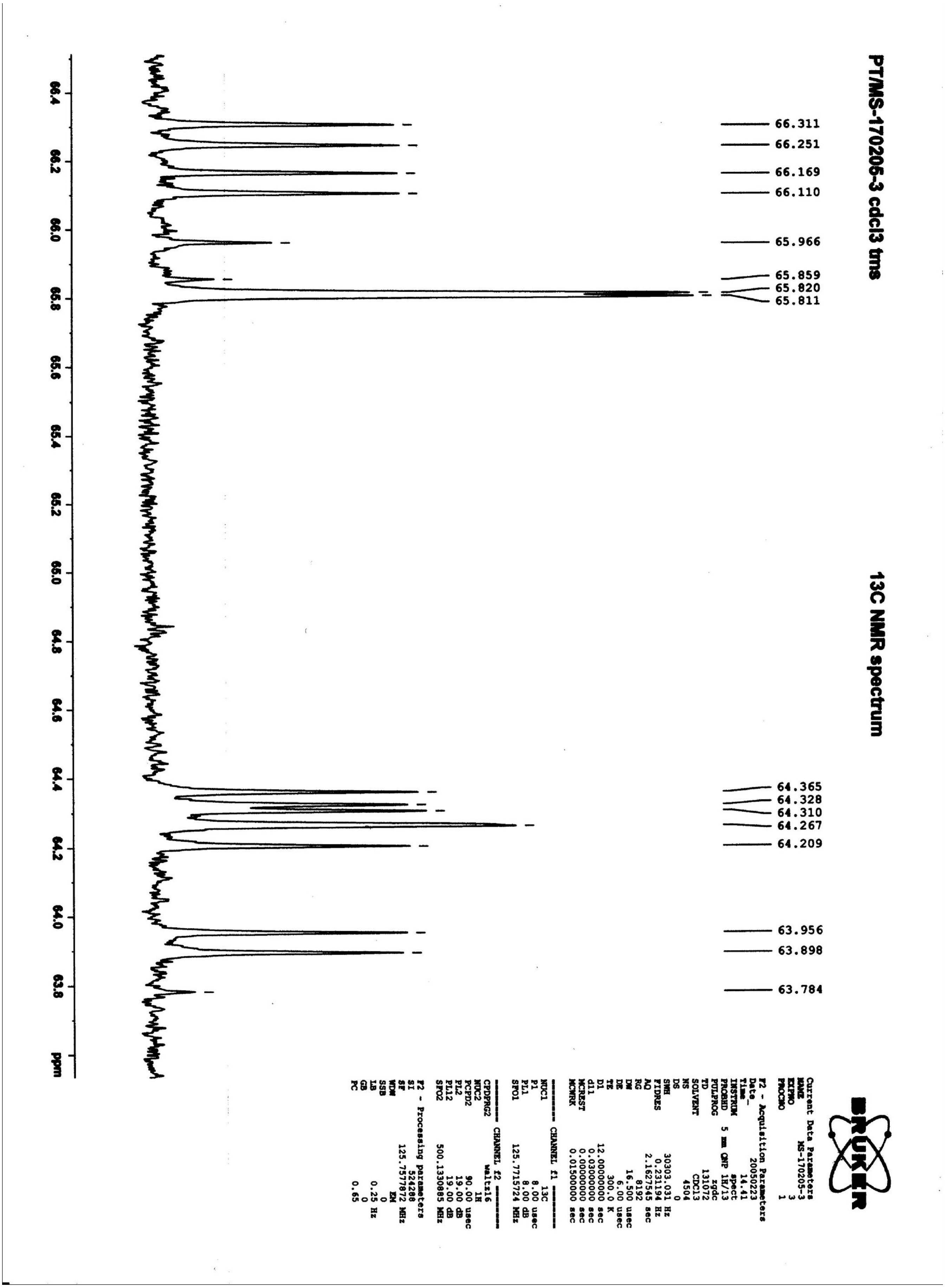
PT/MS-170206-3 cdcl3 tms
13C NMR spectrum
66.311
66.251
66.169
66.110
65.966
65.859
65.820
65.811
64.365
64.328
64.310
64.267
64.209
63.956
63.898
63.784
ppm

Compound **1a**

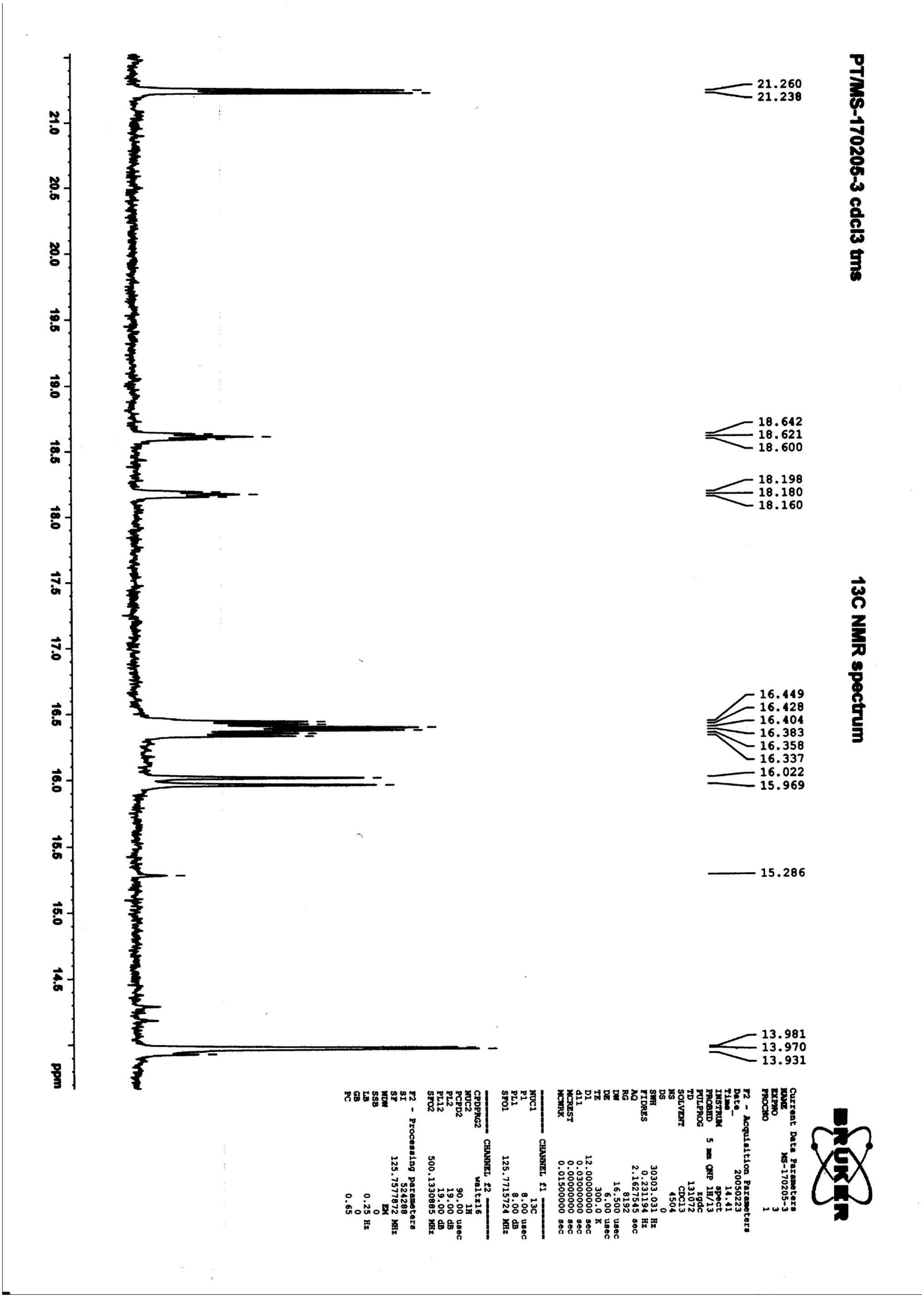
PT/MS-170205-3 cdcl3 tms
13C NMR spectrum
21.260
21.238
18.642
18.621
18.600
18.198
18.180
18.160
16.449
16.428
16.404
16.383
16.358
16.337
16.022
15.969
15.286
13.981
13.970
13.931
ppm
BRUKER

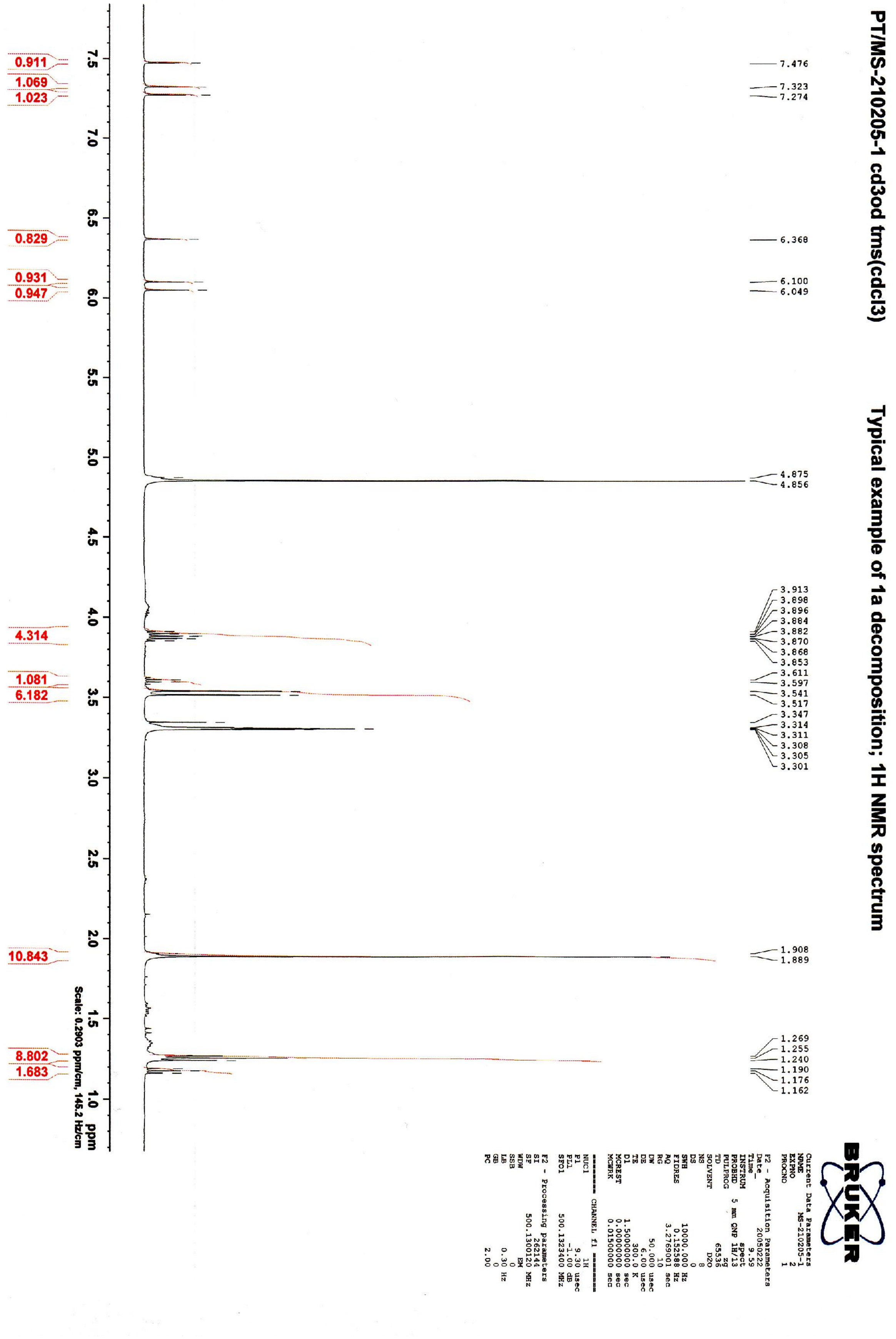
PT/MS-210205-1 cd3od tms(cdcl3)
Typical example of 1a decomposition; 1H NMR spectrum
7.476
7.323
7.274
6.368
6.100
6.049
4.875
4.856
3.913
3.898
3.896
3.884
3.882
3.870
3.868
3.853
3.611
3.597
3.541
3.517
3.347
3.314
3.311
3.308
3.305
3.301
1.908
1.889
1.269
1.255
1.240
1.190
1.176
1.162
0.911
1.069
1.023
0.829
0.931
0.947
4.314
1.081
6.182
10.843
8.802
1.683
ppm
Scale: 0.2903 ppm/cm, 145.2 Hz/cm
BRUKER

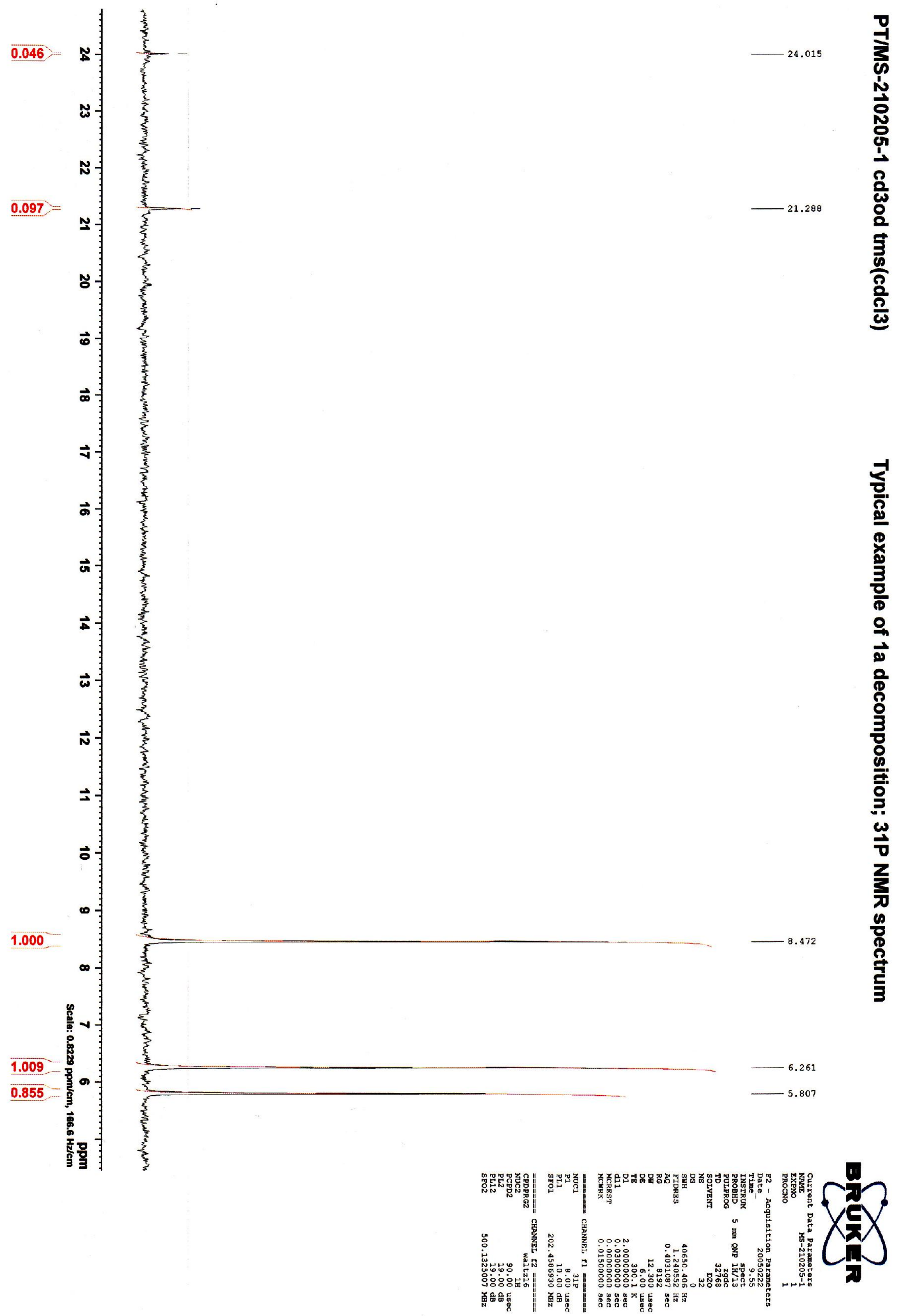
PT/MS-210205-1 cd3od tms(cdcl3)
Typical example of 1a decomposition; 31P NMR spectrum
24.015
21.288
8.472
6.261
5.807
0.046
0.097
1.000
1.009
0.855
ppm
Scale: 0.8229 ppm/cm, 166.6 Hz/cm
BRUKER

PT/MS-190906-2b d2o   **Typical example of 1b decomposition; 1H NMR spectrum**

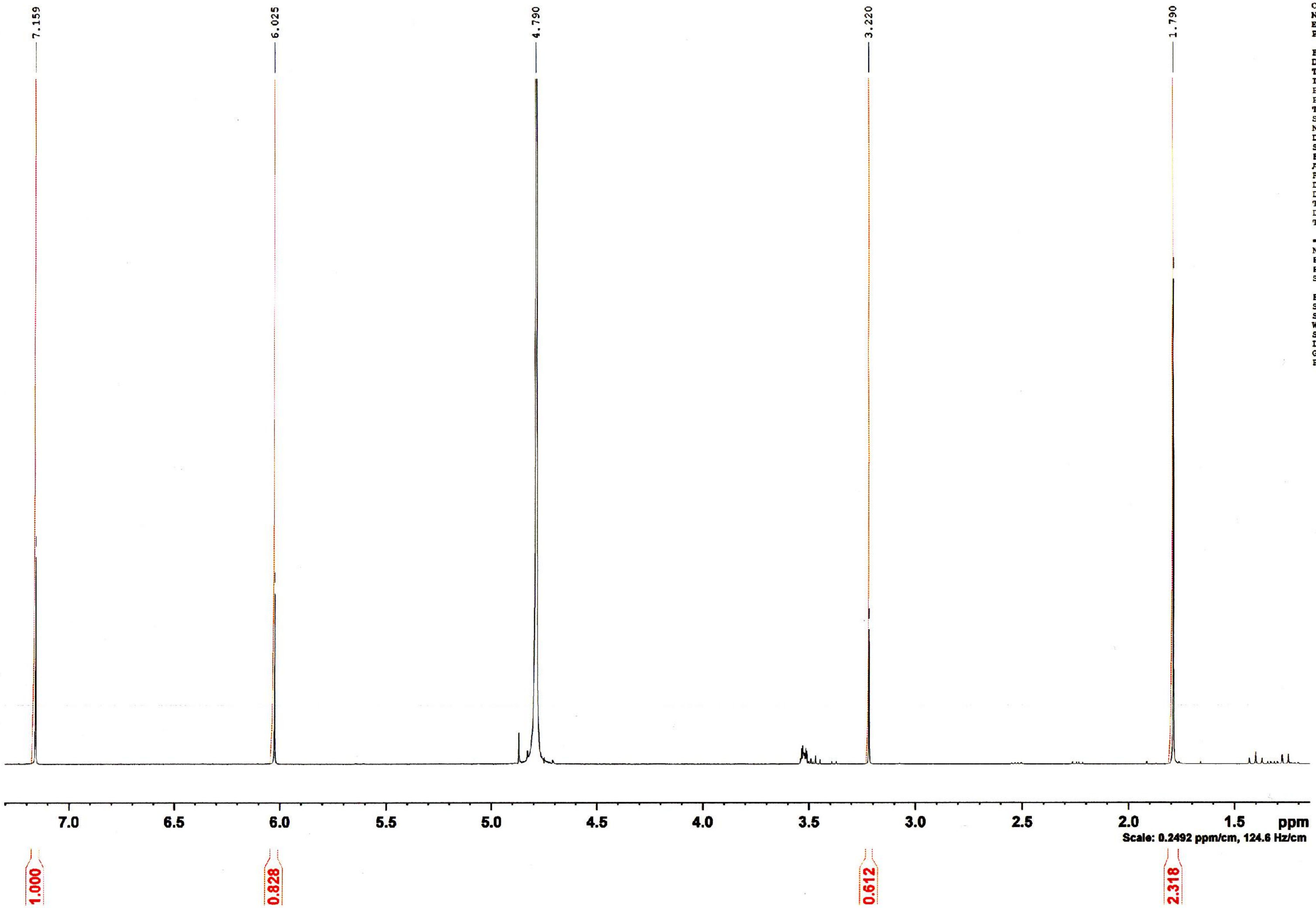

BRUKER

```
Current Data Parameters
NAME          MS-190906-2b
EXPNO                    2
PROCNO                   1

F2 - Acquisition Parameters
Date_             20060919
Time                 13.28
INSTRUM              spect
PROBHD    5 mm QNP   1H/1
PULPROG                 zg
TD                   65536
SOLVENT                D2O
NS                       8
DS                       0
SWH           10000.000 Hz
FIDRES         0.152588 Hz
AQ            3.2769001 sec
RG                      64
DW               50.000 usec
DE                 6.00 usec
TE                293.0 K
D1           1.50000000 sec
TD0                      1

======== CHANNEL f1 ========
NUC1                    1H
P1                  8.75 usec
PL1                -2.00 dB
SFO1       500.1340010 MHz

F2 - Processing parameters
SI                  262144
SF          500.1299637 MHz
WDW                     EM
SSB                      0
LB                    0.30 Hz
GB                       0
PC                    2.00
```

PT/MS-190906-2b d2o Typical example of 1b decomposition; 31P NMR spectrum

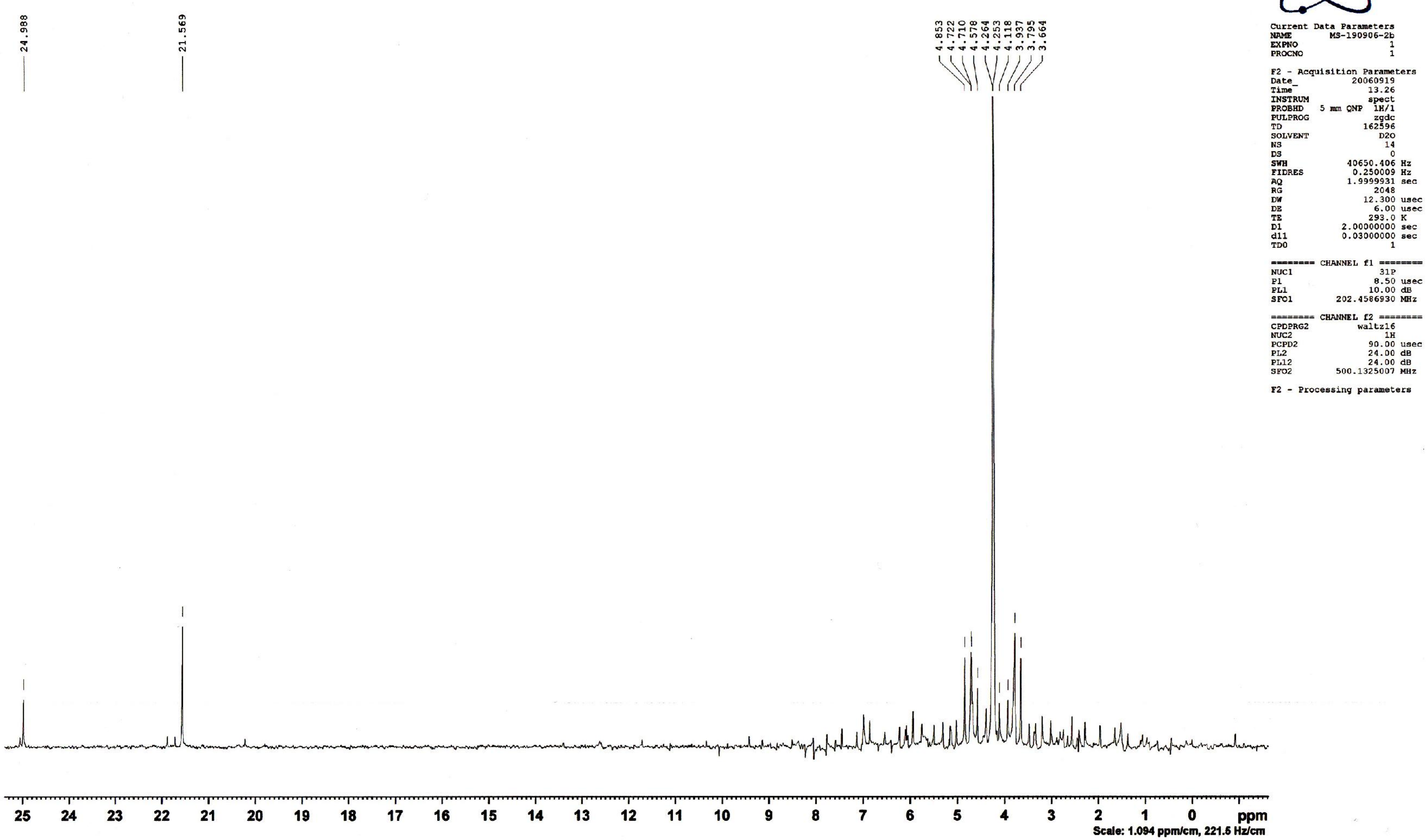

PT/MS-190107-1a d2o Typical example of 1b decomposition in triethyl amine; 1H NMR spectrum

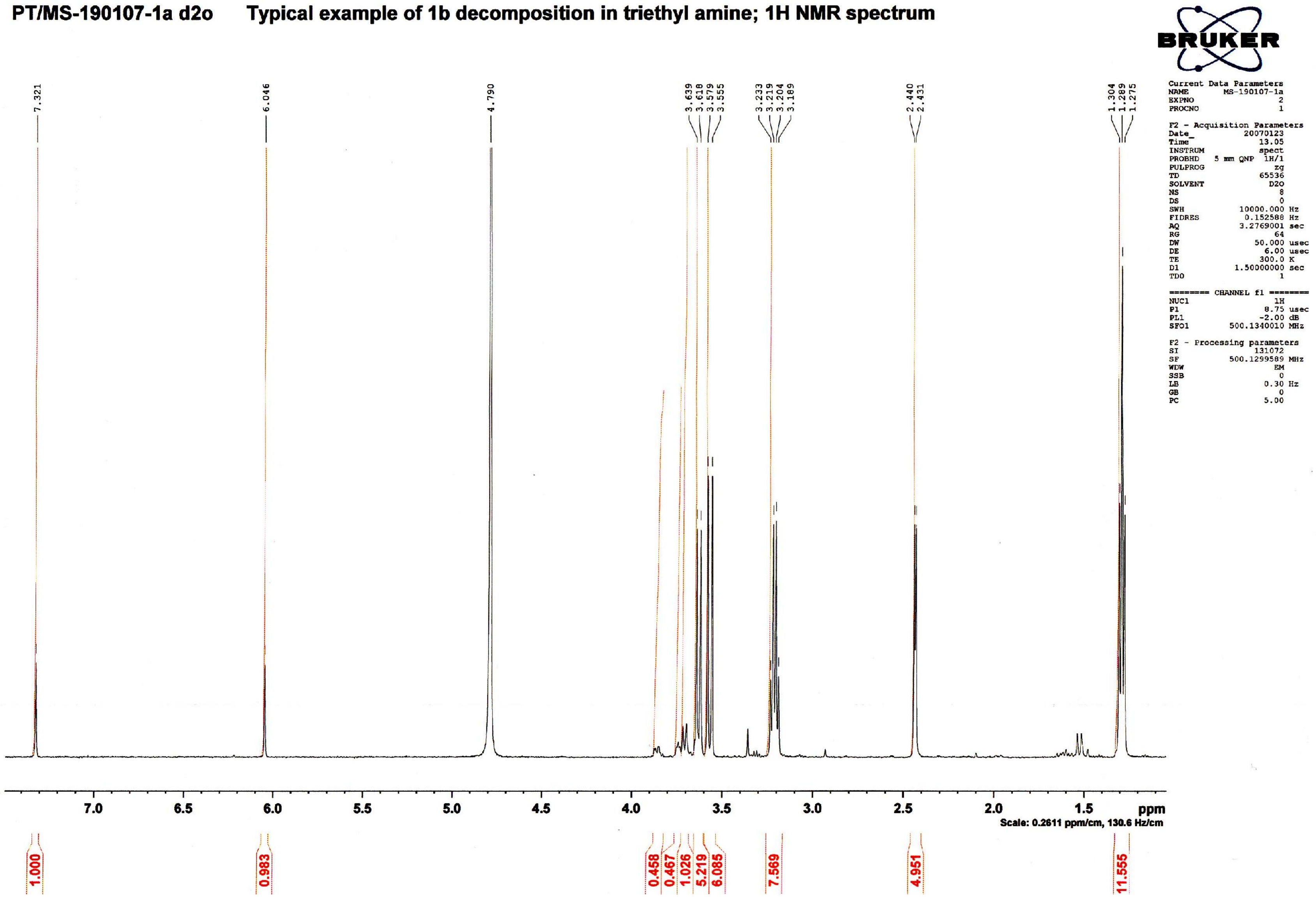

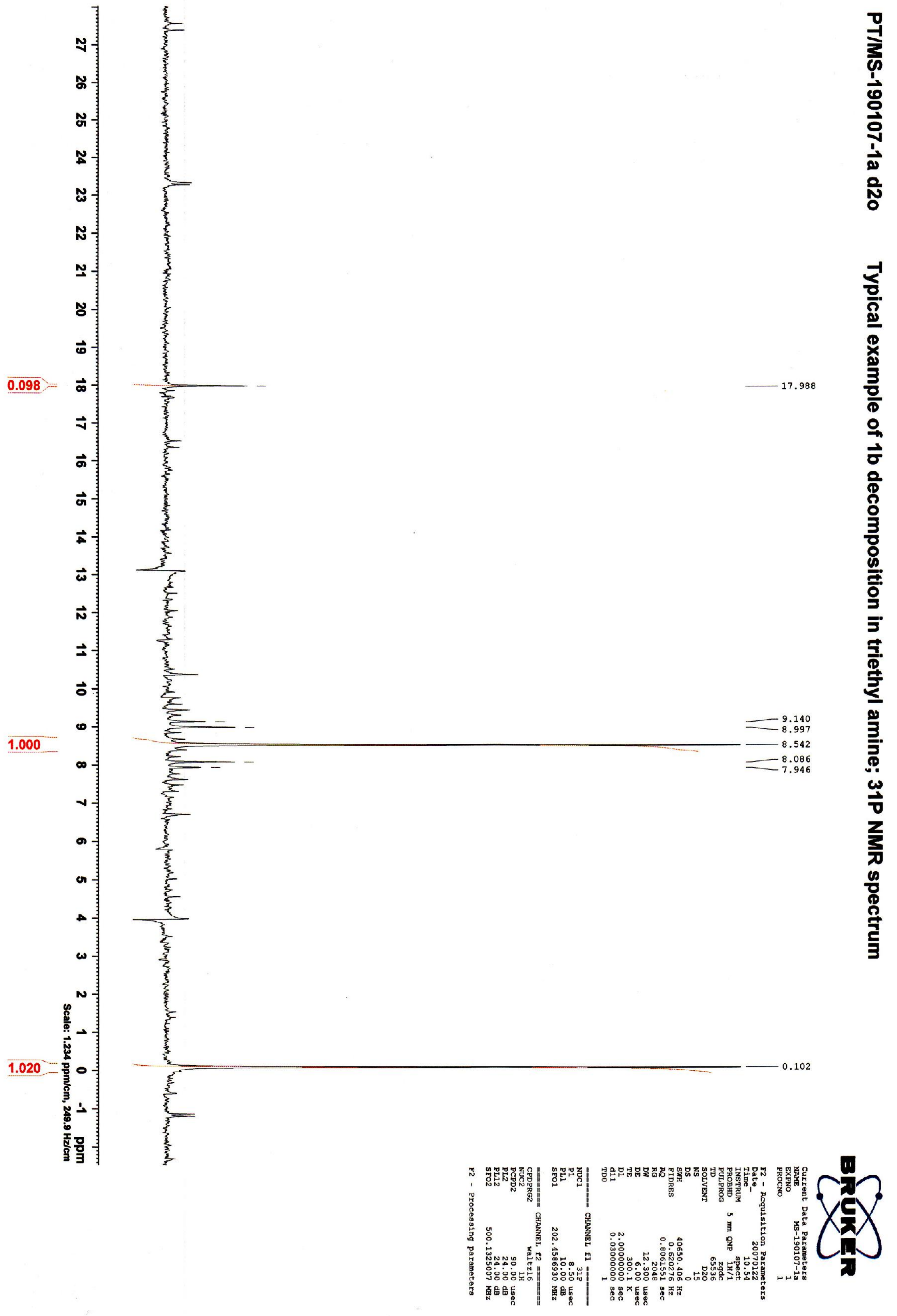
PT/MS-190107-1a d2o
Typical example of 1b decomposition in triethyl amine; 31P NMR spectrum
17.988
9.140
8.997
8.542
8.086
7.946
0.102
0.098
1.000
1.020
27 26 25 24 23 22 21 20 19 18 17 16 15 14 13 12 11 10 9 8 7 6 5 4 3 2 1 0 -1 ppm
Scale: 1.234 ppm/cm, 249.9 Hz/cm
BRUKER
Current Data Parameters
NAME MS-190107-1a
EXPNO 1
PROCNO 1
F2 - Acquisition Parameters
Date_ 20070122
Time 10.54
INSTRUM spect
PROBHD 5 mm QNP 1H/1
PULPROG zgdc
TD 65536
SOLVENT D2O
NS 15
DS 0
SWH 40650.406 Hz
FIDRES 0.620276 Hz
AQ 0.8061551 sec
RG 2048
DW 12.300 usec
DE 6.00 usec
TE 300.1 K
D1 2.00000000 sec
d11 0.03000000 sec
TD0 1
======== CHANNEL f1 ========
NUC1 31P
P1 8.50 usec
PL1 10.00 dB
SFO1 202.4586930 MHz
======== CHANNEL f2 ========
CPDPRG2 waltz16
NUC2 1H
PCPD2 90.00 usec
PL2 24.00 dB
PL12 24.00 dB
SFO2 500.1325007 MHz
F2 - Processing parameters
